# *Lamiophlomis rotata* Identification via ITS2 Barcode and Quality Evaluation by UPLC-QTOF-MS Couple with Multivariate Analyses

**DOI:** 10.3390/molecules23123289

**Published:** 2018-12-11

**Authors:** Jian Wang, Yun-Ling Gao, Yi-Long Chen, Yi-Wen Chen, Yi Zhang, Li Xiang, Zheng Pan

**Affiliations:** 1 College of Traditional Chinese Medicine, Chongqing Medical University, Chongqing 400016, China; wj_2000_abc@163.com; 2 Chongqing Key Laboratory of Traditional Chinese Medicine for Prevention and Cure of Metabolic Diseases, Chongqing 400016, China; 3 School of Bio-information, Chongqing University of Post and Telecommunications, Chongqing 400065, China; gaoyl1@cqupt.edu.cn (Y.-L.G.); chenyw@cqupt.edu.cn (Y.-W.C.); 4 Chongqing Academy of Chinese Materia Medica, Chongqing 404000, China; zychenyl@163.com; 5 College of Ethnic Medicine, Chengdu University of Traditional Chinese Medicine, Chengdu 611130, China; letter2013@sina.com; 6 Institute of Medicinal Plant Development, Chinese Academy of Medical Sciences, Peking Union Medical College, Beijing 100700, China; lxiang@icmmac.cn

**Keywords:** *Lamiophlomis rotata*, ITS2 DNA barcodes, UPLC-QTOF-MS, metabolomics, multivariate analysis

## Abstract

*Lamiophlomis rotata* (*L. rotata*), is known as “Daba” in the Tibetan region, *Ajuga ovalifolia* and *Oreosolen wartii* have also been utilized as substitutes for “Daba”, however, only *L. rotata* has been officially listed in the Chinese Pharmacopoeia for hemostasis preparations. To safely apply the traditional uses of the herb, internal transcribed spacer 2 (ITS2) DNA barcodes were employed to discriminate *L. rotata* from its adulterants. For further evaluation of the quality of different originating habitats, the chemical profiles of 25 samples were determined by ultra-high-performance liquid chromatography coupled with time-of-flight mass spectrometry (UPLC-QTOF-MS) coupled with multivariate analyses. ITS2 DNA barcodes differentiated *L. rotata* from *O. wartii* and *A. ovalifolia* accurately. A neighbor-joining (NJ) tree showed that three origins clustered into three clades. Forty-nine compounds were identified in the total ion current (TIC) profile of *L. rotata*. Additionally, two pairs of isomers were identified for the first time by using mass spectrometry fragmentation. The differences between the variable habitats were determined by multivariate statistical analysis of the UPLC-QTOF-MS data from 25 specimens. Ten compounds were identified as the characteristic markers distinguishing the sample from four geographical origins. The results also suggest that samples from Qinghai and Sichuan province would be the most suitable choice for traditional prescriptions and preparations.

## 1. Introduction

*Lamiophlomis rotata* (Benth.) kudo (*L. rotata*), a plant growing at high altitude in China, has been used to treat rheumatic arthritis and grasserie for more than 2000 years in the Traditional Tibetan System (TTS). It is known as “Daba” and “Dabuba” in the TTS, additionally, *Ajuga ovalifolia* (*A. ovalifolia*) and *Oreosolen wartii* (*O. wartii*) have been utilized as substitutes for Daba in the TTS [1]. However, after extensive clinical use and investigation, only *L. rotata* (Duyiwei in Chinese) was officially listed in the Chinese Pharmacopoeia in 1989 for hemostasis and analgesic preparations [2], such as Duyiwei Capsules, Duyiwei Pills, Qizheng Xiaotong Plaster, etc. The herb has been, and continues to be, used for different therapeutic purposes in various folk medicines. Therefore, a method for simple and accurate identification of *L. rotata* is necessary to assure efficacy and biosafety. A number of publications using morphological, histological, and molecular biological methods for species identification of *L. rotata* or *O. wartii* origin have been reported [3,4,5]. Nevertheless, few of these methods are aimed at distinguishing *L. rotata* from *O. wartii* and *A. ovalifolia*.

Recently, internal transcribed spacer (ITS) DNA barcodes have been developed for the identification of plant species. Among these DNA barcodes, ITS2, being part of ITS, is relatively easy to be amplified using one pair of universal primers, and is one of the best performing barcodes identified for medicinal plants thus far [6,7,8,9]. In the current study, we utilized ITS2 as a DNA barcode to differentiate the three medicinal plants in order to ensure safe application of *L. rotata* in traditional uses.

Raw plant materials used for medicinal products from different geographical regions are often inconsistent in chemical composition [10]. Although ITS2 may distinguish *L. rotata* from its adulterants; this technique failed to assay the variability of bioactive chemical in *L. rotata* from different habitats. Dozens of compounds have been found in the herb, with principal efficiency ingredients being iridoids, flavonoids, and phenylethanoids [11,12,13]. Since 2005, Luteolin and total flavonoids have been used for the quality control of the herb preparations in the Chinese Pharmacopoeia [14]. Consequently, various methods have been developed to qualitatively and quantitatively analyze flavonoids to measure the quality of *L. rotata* and its preparations [15,16]. In 2010, shanzhiside methyl ester and 8-*O*-acetyl shanzhiside ethyl ester were selected as the marker compound for quality control of the herb. Additionally, total flavonoids has also been used to reasonably control the quality of preparations [17]. Therefore, a series of analytical methods have also been developed for quantitative analyses of iridoid glycosides over the last ten years [18,19,20,21]. However, these methods may cause confusion when investigating the similarity and variability in samples from different geographical origins, because of quantification according to different quality standards with one or few marker compounds. In view of the above reasons, it is necessary to achieve comprehensive chemical composition analyses to evaluate the variability in *L. rotata* from different geographical origins.

To achieve the second goal, a method using ultra-high-performance liquid chromatography coupled with time-of-flight mass spectrometry (UPLC-QTOF-MS) was developed, and a metabolomic approach was employed with principal component analysis (PCA) and partial least-squares discriminant analysis (PLS-DA). The UPLC-QTOF-MS data were assayed to characterize components in *L. rotata* from different geographical regions, and to further explore the relationship between different samples to ensure the intended therapeutic effects.

## 2. Results

### 2.1. Measurement of DNA Divergence for ITS2

#### 2.1.1. Sequence and Inter-/Intra-Specific Variation Analysis

ITS2 segments were successfully extracted from all samples. The polymerase chain reaction (PCR) amplification success rates for ITS2 were 100%. All PCR products were successfully sequenced, and high-quality bidirectional sequences were obtained.

The sequence characteristics are summarized in Table 1. The average G-C contents of the ITS2 sequences in *L. rotata* and *A. ovalifolia* were 70% to 72% and 65%. ITS2 sequences of *L. rotata* ranged from 0 bp to 219 bp with five variable sites; five haplotypes were identified from 25 samples. ITS2 sequences of *A. ovalifolia* ranged from 0 bp to 229 bp with three variable sites, and three haplotypes were identified. In general, gene segments are available when the minimum inter-specific distances are larger than the maximum intra-specific distances by the Kimura 2-Parameter (K2P) model. In this experiment, the minimum inter-specific distances were 0.258 cM, and the maximum intra-specific distances were 0.021 cM (Table 1). Therefore, the ITS2 region could be an ideal barcode for discriminating the three origins of *L. rotata*, *A. ovalifolia*, and *O. wartii*.

#### 2.1.2. Neighbor-Joining (NJ) Tree Analysis

**Figure 1:**
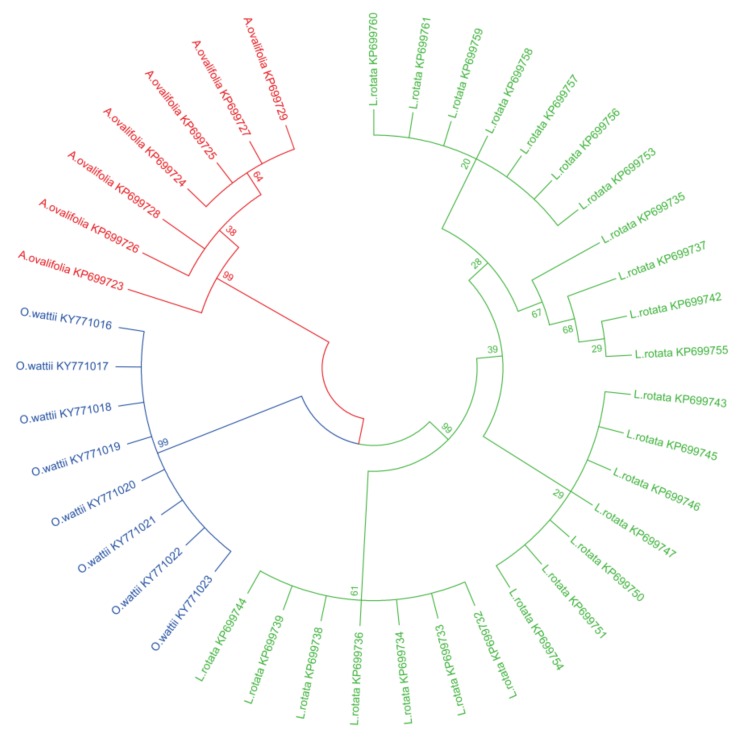
Neighbor-joining trees of *L. rotata*, *A.ovalifolia*, and *O.wartii* by internal transcribed spacer 2 (ITS2). The bootstrap scorers (1000 replicates) are shown (≥50%) for *L. rotata* and its adulterants.

In this study, a phylogenetic tree was constructed using the neighbor-joining (NJ) method, with 1000 bootstrap replicates for ITS2 regions (Figure 1). All species were clearly identified, including the medicinal and non-medicinal species. Each of the three species is in one branch of the phylogenetic tree. Specifically, the NJ tree also showed that all samples of *L. rotata* were clustered into three subgroups according to their geographical origins. The samples from Tibet, and Gansu, are gathered into one branch each and the samples of Sichuan and Qinghai are gathered together into another branch.

### 2.2. Identification of the Constituents in L. rotata by UPLC-QTOF-MS Spectra

**Figure 2:**
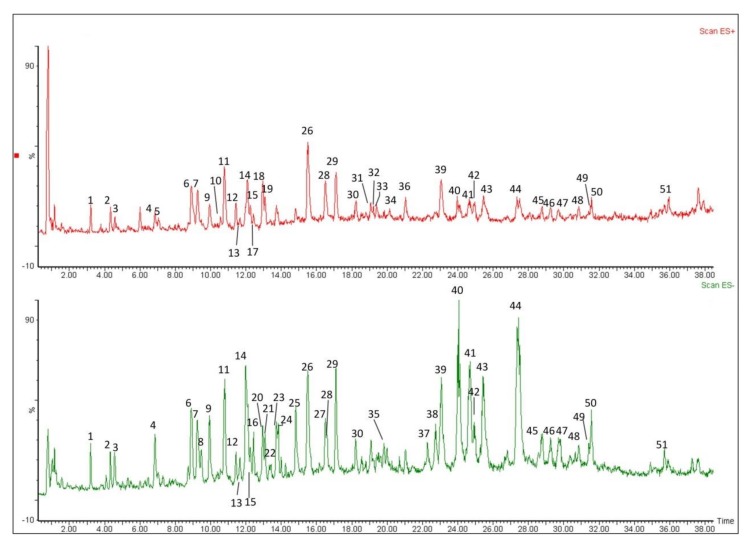
Total ion current chromatograms of substances in the extract of *L. rotata*, under positive and negative ionization modes.

Crude extracts of *L*. *rotata* were analyzed by mass and MS^n^ in negative and positive ion modes (Figure 2). The SciFinder Scholar and PubChem data bases were searched for the spectral data of compounds reported previously in the *L. rotata* and *Lamium* species [22,23,24,25,26,27], and a total of 51 compounds in four classes were detected in the total ion current (TIC) profile of *L. rotata*, and 49 of these were identified by comparing the retention times and mass spectra of the compounds to those of authentic standards, including 23 iridoids, 16 phenylethanoid glycosides, nine flavanoids and one phenolic acid (Table 2).

**Figure 3:**
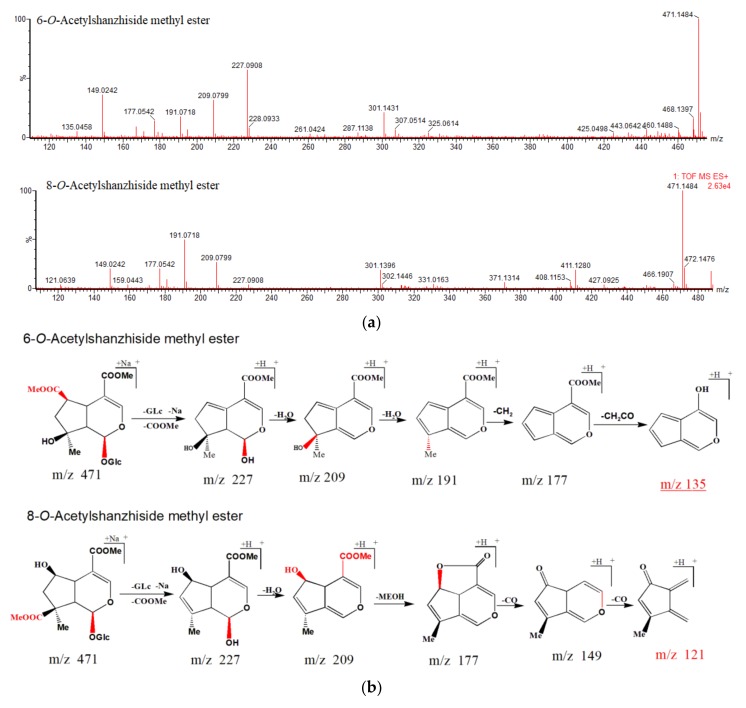
(**a**). MS^2^ spectra of 8-*O*-acetylshanzhiside methyl ester and 6-*O*-acetylshanzhiside methyl ester. (**b**) Possible mass fragmentation pathways of 8-*O*-acetylshanzhiside methyl ester and 6-*O*-acetylshanzhiside methyl ester.

For the first time, two pairs of isomers (8-*O*-acetylshanzhiside methyl ester and 6-*O*-acetylshanzhiside methyl ester, phlorigidoside C, and zaluzioside) were identified by mass spectrometry based on their different group substitution positions. Peaks 19 and 29 exhibited the same [M + Na]^+^ ions at *m*/*z* 471 in the positive mode, consistent with a molecular formula of C_19_H_28_O_12_, they both product the ions at *m*/*z* 227 and 209 Da. However, a hydroxyl group was linked to C-6 in compound 29, so it easily lost a methanol molecule to form a lactone with the carboxymethyl (COOCH_3_) group at the C-4 position. This came, with a (neutral) loss of 32 Da, and successive losses of two CO groups, which yielded further peaks simultaneously appearing at *m*/*z* 177 Da (*m*/*z* 209→177, Δ*m* = 32 Da, MeOH group loss), 149 Da (*m*/*z* 177→149, Δ*m* = 28 Da, CO group loss), and the characteristic ion at *m*/*z* 121 Da (*m*/*z* 149→121, Δ*m* = 28 Da, CO group loss). Since there was no hydroxyl group substituted at the C-6 position in compound 19, after successive losses of H_2_O, CH_2_, and CH_2_O groups, the peak yielded ions at *m*/*z* 191 Da (*m*/*z* 209→191, Δ*m* = 18 Da, H_2_O group loss), 177 Da (*m*/*z* 191→177, Δ*m* = 14 Da, CH_2_ group loss), and the distinctive ion at m/z 135 Da (*m*/*z* 177→135, Δ*m* = 42 Da, CH_2_CO group loss). The proposed fragmentation pathways of the isomers are shown in Figure 3a, b.

**Figure 4:**
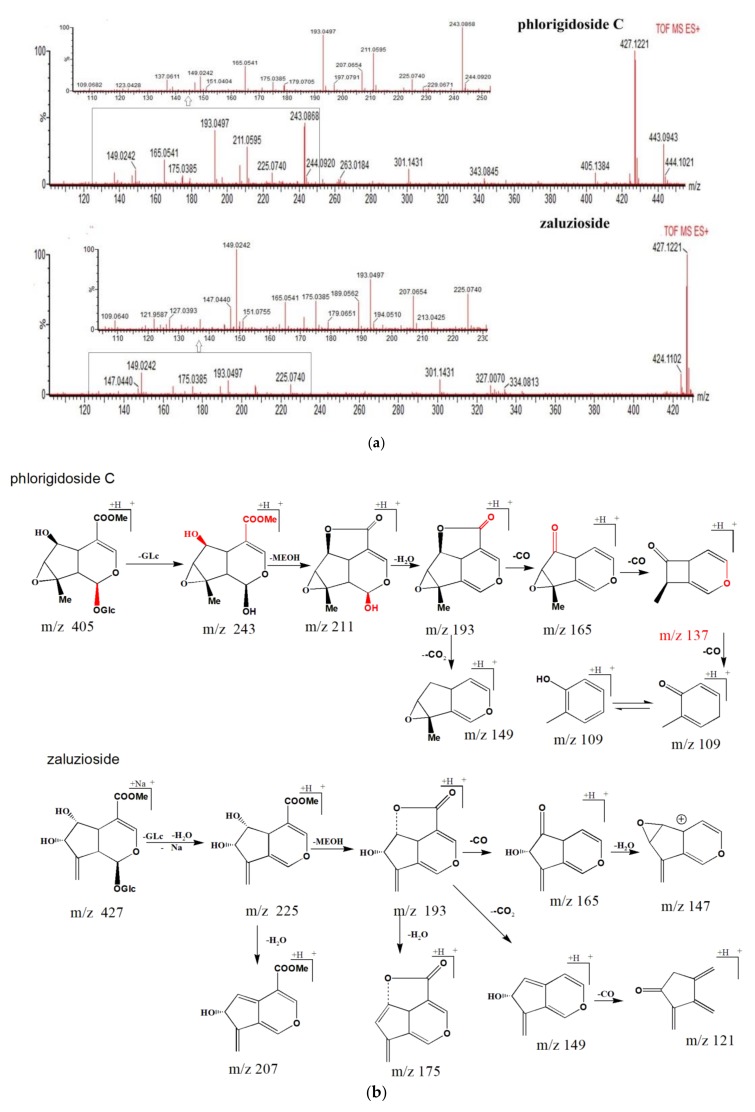
(**a**) MS^2^ spectra of phlorigidoside C and zaluzioside. (**b**) Possible mass fragmentation pathways of phlorigidoside C and zaluzioside.

Peaks 9 and 33 exhibited the same molecular formula of C_19_H_28_O_12_ with losses of 162 Da (glucose unit), 32 Da (methanol unit), and 18 Da (H_2_O unit), the pair of isomers produced the same ion at *m*/*z* 193 Da. For peak 9, the compound successively lost three CO groups, the peak yielded ions at *m*/*z* 165 Da (*m*/*z* 193→165, Δ*m* = 28 Da, CO group loss), and distinctive ions at *m*/*z* 137 Da (*m*/*z* 165→137, Δ*m* = 28 Da, CO group loss), 109 Da (*m*/*z* 137→109, Δ*m* = 28 Da, CO group loss). With the 5,6-di-OH substituted into compound 33, the ion at *m*/*z* 149 Da (*m*/*z* 193→149, Δ*m* = 44 Da, CO_2_ group loss) and the characteristic ion at *m*/*z* 121 Da (*m*/*z* 149→121, Δ*m* = 28 Da, CO group loss), was observed in peak 33. Additionally, the compound provided characteristic ions at *m*/*z* 147 Da (*m*/*z* 193→165, Δ*m* = 28 Da, CO group loss; *m*/*z* 165→147, Δ*m* = 18 Da, H_2_O group loss). The proposed fragmentation pathway of phlorigidoside C and zaluzioside are shown in Figure 4a, b. Furthermore, 6-*O*-β-d-glucopyranosyl shanzhiside, leucosceptoside A, phlorigidoside A/phlorigidoside B, and forsythoside C/campneoside II, are reported for the first time in MS metabolomics profiles of *L. rotata*.

### 2.3. Multivariate Statistical Analysis

**Figure 5:**
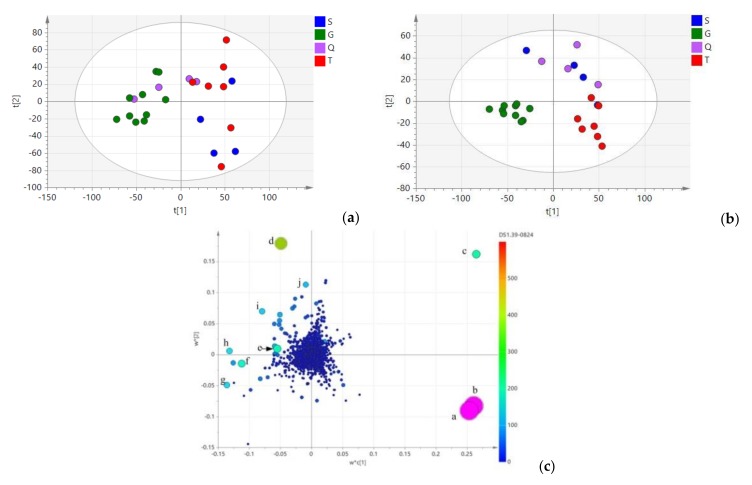
(**a**): Principal component analysis (PCA) score plot of *L. rotata* in four geographical origins. (**b**): Partial least-squares discriminant analysis (PLS-DA) score plot of *L. rotata* in four geographical origins. (**c**): Loading plot of PLS-DA analysis of *L. rotata*. a: forsythoside B (*t_R_* 24.04 min, *m*/*z* 755.1098), b: verbascoside (*t_R_* 24.04 min, *m*/*z* 623.0175), c: Kaempferol-3-glycoside (*t_R_* 13.83 min, m/z 593.1594), d: Luteolin-7-*O*-*β*-d-glucopyranside (*t_R_* 24.65 min, *m*/*z* 447.1927), e: Chlorogenic acid (*t_R_* 4.59 min, *m*/*z* 353.0718), f: Phlorigidoside C (*t_R_* 9.36 min, *m*/*z* 427.1621), g: Loganin (*t_R_* 21.02 min, *m*/*z* 413.1342), h: 5-deoxy-pulchelloside I (*t_R_* 11.99 min, *m*/*z* 429.0361), i: 7-epi-loganin (*t_R_* 18.23 min, *m*/*z* 412.9987), j: Decaffeoylcrenatoside (*t_R_* 14.26 min, *m*/*z* 459.2102).

Principle component analysis (PCA) was used to maximize the discrimination and present the metabolite differences among groups (Figure 5a), the result demonstrated that 25 samples were separated into two groups; all samples from Gansu Province and two samples from Qinghai Province were clustered into group Ⅰ, other samples were clustered into group Ⅱ. Moreover, the 13 samples of group Ⅱ were branched into two subgroups. Partial least-squares discriminant analysis (PLS-DA) was also used for a better understand of the geographical origins of the collected samples. As shown in Figure 5b, all of the *L. rotata* samples were clustered into three groups. The high R^2^Y (0.814) of this model presented a goodness of fit, and the Q2 at 0.316 indicated good predictivity. The samples of group Ⅰ were from Gansu Province (green dots), whereas group Ⅱ was composed of samples from Tibet (red dots). Notably, the samples from Qinghai and Sichuan province (purple dot and blue dots, respectively) forming group Ⅲ, could not be distinguished from each other, this is because, these populations come from a similar natural environment, which it was referred to as the “Amdo Tibetan area” during the Qing Dynasty.

As shown in the loading and score plots of serum different serum (Figure 5c), there were ten biomarkers found to characterize samples from the four geographical origins. According to their significance in discriminating geographical characteristics, these compounds were identified as a (forsythoside B, *t_R_* 24.04 min, *m*/*z* 755.1098), b (verbascoside, *t_R_* 24.04 min, *m*/*z* 623.0175), c (kaempferol-3-glycoside, *t_R_* 13.83 min, *m*/*z* 593.1594), d (phlorigidoside C, *t_R_* 9.36 min, *m*/*z* 427.1621), e (chlorogenic acid, *t_R_* 4.59 min, *m*/*z* 353.0718) f (loganin, *t_R_* 21.02 min, *m*/*z* 413.1342), g (luteolin-7-*O* -*β*-d-glucopyranside, *t_R_* 24.65 min, *m*/*z* 447.1927), h (5-deoxypulchelloside I, *t_R_* 11.99 min, *m*/*z* 429.0361), i (7-epi-loganin, *t_R_* 18.23 min, *m*/*z* 412.9987), and j (decaffeoylcrenatoside, *t_R_* 14.26 min, *m*/*z* 459.2102). It is also clearly shown that samples from Tibet are characterized by a high content of phenylethanoid glycosides compound a and b, but a low content of iridoid and flavonoid glycosides. Samples from the Gansu location had a higher relative concentration of compound g and f, and a low content of phenylethanoid and flavonoids glycosides. Similarly, samples from the Qinghai and Sichuan province were branched into one group, and are characterized by a high content of compound c, d, e, h, i, and j. The results also suggest that these samples are characterized by a high content of flavonoids glycosides, a moderate content of iridoid glycosides, and a low content of phenylethanoid glycosides. Since iridoid glycoside and total flavonoids contents are used to qualitatively and quantitatively analyze *L. rotata* and the preparations in the latest Chinese Pharmacopoeia [28], the above result shows that, samples from the Qinghai and Sichuan provinces would be the most suitable choice for traditional prescriptions and preparations.

## 3. Discussion

Liu et al. reported the detection of *L. rotata* from three genetic groups corresponding to three geographic regions using inter simple sequence repeats (ISSR) and randomly amplified polymorphic DNA (RAPD) techniques [3]. Pan et al. investigated the systematic positions of *Lamiophlomis* and *Paraphlomis* (Lamiaceae) based on ITS DNA barcodes and chloroplast *rpl16* and *trn L-F* sequences [4]. In this paper, the variation at the genus and species level among *L. rotata*, *O. wartii* and *A. ovalifolia* was distinguished for the first time using ITS2 DNA barcodes, the result confirmed that ITS2 DNA barcodes are one of the best-performing barcodes for identifying medicinal plants characteristics.

In our previous study, we reported a method using ^1^H nuclear magnetic resonance (NMR) spectroscopy with multivariate analysis to discriminate the extracts in deuterium reagents of *L. rotata* from the Gansu, Tibet, and Qinghai provinces. The result of that study revealed that the Gansu samples had a higher iridoid glycoside contents, and the Tibet samples had a high content of phenylethanoid glycosides. However, as NMR spectroscopy lacks sensitivity, compounds present at low levels were not analyzed in previous studies, but were found by the UPLC-QTOF-MS method. Trace compounds, such as, 5-deoxypulchelloside I, kaempferol-3-glycoside, and 7-epi-loganin, have been found to characterize samples of four geographical origins, a comprehensive chemical composition profile of 25 samples were revealed, and the result also confirmed that of our previous study, that Tibetan samples had higher content of phenylethanoid glycosides while individuals from the Gansu province had a higher iridoid glycoside contents. In this study, variation was seen in the overall pattern of metabolites between samples from different geographical locations. Duan et al. also found that growth locations have greater impact on the metabolite composition and quantity than the genotypes (cultivated versus wild) in Menggu Huangqi (*Astragalus mongholicus*) [29]; Huang et al. reported that the chemical level and composition of *Cistanche deserticola* was affected by the key factors of temperature, moisture, and illumination [30]. Additionally, samples from the Sichuan provinces were employed for a more comprehensive origin study than that undertaken in our previous studies. Altogether, this study contributes to closing knowledge gaps in the topic of systematic characterization of *L. rotata* and its safe application in traditional uses.

## 4. Materials and Methods

### 4.1. Plant Materials, Reagents, and Chemicals

Twenty-five populations of *L. rotata* were collected from throughout the geographical distribution of official source plants, including the Tibet, Qinghai, Sichuan, and Gansu provinces (Table 3). The sampling strategy covered most of its presently known populations [29]. Eight individual samples of *A. ovalifolia* were also collected from Qinghai and Gansu provinces, and eight ITS2 sequences of *O. wartii* were downloaded from Gen Bank. Details of the Gen Bank accession numbers, haplotypes of ITS2 sequences, and the locations of the sampling areas are provided in Table 3. The voucher samples were deposited in the College of Ethnic Medicine (Chengdu University of Traditional Chinese Medicine, Chengdu, China) and the Chongqing Academy of Chinese Materia Medica (Chongqing, China). We included all sequences in the final analysis.

Decaffeoylcrenatoside, verbascoside, forsythoside B, luteolin-7-*O*-*β*-d-glucopyranside, shanzhiside methyl ester, 8-*O*-acetylshanzhiside methyl ester, sesamoside, 7,8-dehydro- penstemoside, phlorigidoside C, 7-epi-phlomiol, loganin, and phloyoside II, were purified from *L. rotata* in our laboratory and identified by direct comparison of their ^1^H-NMR and ^13^C-NMR spectra to those in the literature [13,20,21,22,23]; all purities were determined to be >95% by high performance liquid chromatography.

HPLC-grade methanol and formic acid were purchased from Merck (Darmstadt, Germany) and Tedia (Fairfield, OH, USA). Deionized water was prepared using a Millipore water treatment system (Bedford, MA, USA). All other reagents were of analytical grade.

### 4.2. DNA Barcoding: DNA Extraction, PCR Amplification and Sequencing

DNA extraction was performed according to the method described in reference [31]. In brief, samples taken from dried stems of *L. rotata* and *A. ovalifolia* (30 mg) were rubbed for 2 min at a frequency of 30 r/s. DNA was extracted using a Plant Genomic DNA Kit (Tiangen Biotech Co., Beijing, China) in accordance with the manufacturer’s instructions. PCR was carried out according to the following program: 94 °C for 5 min followed by 40 cycles of 94 °C for 30 s, 56 °C for 30 s, and 72 °C for 45 s with DNA polymerase (Biocolor BioScience & Technology Co., Shanghai, China). ITS2-specific primers were used as follows: GTTATGCATGAACGTAATGCTC (5ʹ–3ʹ) as the forward primer and CGCGCATGGTGGATTCACAATCC (5ʹ–3ʹ) as the reverse primer. PCR products were separated and detected using 1% agarose gel electrophoresis. PCR products were purified following the manufacturer’s protocol and directly subjected to sequencing.

The PCR products were visualized on agarose gels (the electrophoresis was run in 1 × TBE for 20 min at a constant voltage 120 V). After electrophoresis, purified PCR products were bidirectionally sequenced using the same primers as were used for PCR in a 3730XL sequencer (Applied Biosystems, Foster, CA, USA).

### 4.3. Sequence Alignment and Analysis

ITS2 sequences of *O. wartii* were collected from the Gen Bank database. Sequences attained from sequencing of the samples were submitted to Gen Bank database (Table 3).

Proofreading and coting assembly of the sequencing peak diagrams was performed using Codon Code Aligner 3.7.1 (Codon Code Co., Centreville, MA, USA). The ITS2 region was obtained using the annotation method based on the Hidden Markov model（HMMer） to remove the 5.8S and 28S sections at both ends of the sequences. All sequences were aligned (MUSCLE option) by MEGA 6.0 (Center for Evolutionary Medicine and Informatics, Tempe, AZ, USA), and the genetic distances were calculated according to the K2P model. The distribution of intra-versus/inter-specific variability was assessed by DNA barcoding gaps. An NJ tree was constructed and bootstrap resampling (1000 replicates) was conducted to assess the confidence in the phylogenetic analysis by MEGA 6.0 [32].

### 4.4. Sample Preparation

Dried stems samples of *L. rotata* (1.0 g of powder each) were extracted into 10 mL of 70% aqueous methanol in an ultrasonic bath for 30 min and cooled at room temperature. The extraction was repeated three times using fresh aliquots of the solvent. After combining the three aliquots, the solutions were centrifuged at 12,000 rpm for 10 min and filtered through 0.22-μm pore membranes prior to UPLC-QTOF-MS analysis.

### 4.5. UPLC-QTOF-MS Conditions

Analyses were performed with a Waters Acquity UPLC system (Waters, Milford, MA, USA) equipped with a binary solvent delivery system, an auto-sampler, and photodiode-array detection (DAD) system. The column was a Waters Acquity UPLC BEH C18 column (100 mm × 2.1 mm, 1.8 μm particle size). The mobile phases were (a) water with 0.1% (*v*/*v*) formic acid and (b) methanol with 0.1% (*v*/*v*) formic acid. The optimized elution conditions were as follows: Holding at 7% B for 1 min; a linear gradient from 7% to 11% B (all *v*/*v*) (1 to 4 min), 11% to 13% B (5 to 10 min), 13% to 19% B (15 to 19 min), 19% to 31% B (19 to 24 min), 31% to 45% B (24 to 29 min), 45% to 55% B (29 to 35 min), 55% to 100% B (35 to 36 min), isocratic 100% B for 1 min, and then back to 7%) B in 1 min. The flow rate was 0.3 mL/min. The column temperature was 35 °C. The injection volume was 2 μL.

Mass spectrometry data were obtained using a Xevo G2 Q/TOF (Waters MS Technologies, 129 Manchester, UK) fitted with an electron spray ionization source. Each sample was analyzed twice, once in positive ionization mode and once in negative ionization mode. MS full scans were acquired mode over the range (*m*/*z*) 100 to 1000 Da in two channels with a scan time of 1 s. The capillary voltages were set to 2500 V and the cone voltage to 40 V [33]. Nitrogen gas was used both as a nebulizer and for desolvation. The desolvation and cone gas flow rates were 650 and 50 L·h^−1^, respectively. The desolvation temperature was 300 °C, and the source temperature was 100 °C, the capillary voltage and cone voltage were set to 2700 V and 35 V. The Leu-Enkephalin ions at *m*/*z* 556.2771 and 554.2615 were used to calibrate the mass accuracy.

### 4.6. Data Processing and Statistical Analysis

The original data were processed for alignment, data reduction, and normalization by Marker Lynx software (Waters, Manchester, UK), and the processed data were exported to SIMCA-P software (ver. 13.0; Umetrics, Umeå, Sweden) for data analysis. A list of the intensities of detected peaks was generated using the retention time (*t_R_*) and the mass data (*m*/*z*) pairs to identify each peak. An arbitrary ID was assigned to each *t_R_*–*m*/*z* pair in the order of their UPLC elution to facilitate data alignment. This procedure was repeated for each run. Ions from different samples were considered to be identical when they had the same *t_R_* (tolerance within 0.01 min) and *m*/*z* (tolerance within 0.01 Da). If a peak was not detected in a particular sample, that ion intensity was recorded as zero. In multivariate analysis of statistical significance, *p* < 0.05 and variable importance for projection (VIP) > 3, respectively, were set as the screening criteria for potential markers responsible for the discrimination of different groups.

## 5. Conclusions

In summary, using ITS2 DNA barcodes, *L. rotata* was accurately differentiated from *O. wartii* and *A. ovalifolia*. Additionally, an NJ tree showed that all samples of *L. rotata* were clustered into three subgroups according to their geographical origins. For further evaluation of the quality of the herbs from different habitats, a method coupling UPLC-QTOF-MS with multivariate analysis was implemented. Ten compounds were identified as the characteristic markers distinguishing samples from the four geographical origins. The results also suggest that samples from Qinghai and Sichuan province would be the most suitable choice for traditional prescriptions and preparations.

## Figures and Tables

**Table 1:** The Sequence information and intra/inter-specific genetic distance of internal transcribed spacer 2 (ITS2).

Species	Length (bp)	GC Content (%)	Intraspecific Distance	Interspecific Distance cM (mean)
*L. rotata*	219	70–72	0–0.021	0.258–0.286
*A. ovalifolia*	229	65	0–0.016	0.262–0.286
*O. wartii*	208	63	0	0.258–0.350

**Table 2:** The Identification constituents of the constituents in *L. rotate* by ultra-high-performance liquid chromatography coupled with time-of-flight mass spectrometry (UPLC-QTOF-MS) spectra.

Peak Number	RT (min)	Compound	Formula	Calculated (Da)	Selected Ion	Precursor Ion (Da)	Error (ppm)
1	3.212	7-epi-Phlomiol ^b^	C_17_H_26_O_13_	438.1373	[M + Na]^+^	461.2183	1.98
2	4.339	Schismoside ^a^	C_17_H_26_O_12_	422.1424	[M + Na]^+^	445.1398	0.17
3	4.585	Chlorogenic acid ^c^	C_17_H_22_O_8_	354.1315	[M − H]^−^	353.0718	−1.47
4	6.855	Lamalbide ^a^	C_17_H_26_O_12_	422.1424	[M + Na]^+^	445.2325	2.25
5	7.031	Phlomiol/Phloyoside I ^a^	C_17_H_26_O_13_	438.1373	[M + Na]^+^	461.1375	0.23
6	8.890	Shanzhiside ^a^	C_16_H_24_O_11_	392.1319	[M + Na]^+^	415.1109	−0.26
7	9.284	Penstemoside ^a^	C_17_H_26_O_11_	406.1475	[M + Na]^+^	429.1482	0.25
8	9.478	Luteolin-7-glucuronide ^a^	C_21_H_18_O_12_	462.0798	[M − H]^−^	461.1719	2.17
9	9.935	Phlorigidoside C ^a^	C_17_H_24_O_11_	404.1319	[M + H]^+^	405.1384	3.05
10	10.552	Unknown	C_17_H_24_O_11_	404.1319	[M + Na]^+^	427.0109	−2.59
11	10.798	Shanzhiside methyl ester ^b^	C_17_H_26_O_11_	406.1475	[M + Na]^+^	429.1969	1.39
12	11.432	Lamiridoside ^a^	C_17_H_24_O_12_	420.1268	[M + Na]^+^	443.0158	−2.28
13	11.652	6’-*O*-*β*-d-glucopyranosylshanzhiside ^c^	C_22_H_34_O_16_	554.18	[M + Na]^+^	577.3804	3.65
14	11.986	5-Deoxypulchelloside I ^a^	C_17_H_26_O_11_	406.1475	[M + Na]^+^	429.0361	−2.36
15	12.012	Deoxypulchelloside I ^a^	C_17_H_26_O_11_	406.1475	[M + Na]^+^	429.1957	0.72
16	12.268	Kaempferol-3’methyl ^a^	C_16_H_12_O_6_	300.0634	[M − H]^−^	299.1031	1.59
17	12.426	7,8-Dehydropenstemoside ^a^	C_17_H_24_O_11_	404.1319	[M + Na]^+^	427.1821	1.41
18	12.461	5-Desoxylamiide ^a^	C_17_H_26_O_11_	406.1475	[M + Na]^+^	429.1239	−0.32
19	12.945	6-*O*-Acetylshanzhiside methyl ester ^b^	C_19_H_28_O_12_	448.1581	[M + Na]^+^	471.1484	1.01
20	13.324	Rossicaside C/Rossicaside D ^a^	C_30_H_36_O_14_	620.2105	[M − H]^−^	619.1026	−2.27
21	13.421	Lagotoside B/Lagotoside C ^a^	C_17_H_22_O_8_	354.1315	[M − H]^−^	353.1308	−1.60
22	13.729	Rossicaside D/Rossicaside C ^a^	C_30_H_36_O_14_	620.2105	[M − H]^−^	619.1036	−1.61
23	13.834	Kaempferol-3-glycoside ^a^	C_30_H_26_O_13_	594.1373	[M − H]^−^	593.1594	0.51
24	13.993	Decaffeoylcrenatoside ^a^	C_20_H_28_O_12_	460.1581	[M − H]^−^	459.2102	0.21
25	14.257	Seguinoside E ^a^	C_25_H_30_O_14_	554.1636	[M − H]^−^	553.1957	1.31
26	15.515	Phloyoside II ^b^	C_17_H_25_ClO_12_	456.1034	[M + Na]^+^	479.1851	1.91
27	16.483	Unknown	C_16_H_24_O_12_	408.1267	[M + Na]^+^	431.1171	0.90
28	16.571	Chlorotuberroside ^a^	C_17_H_25_ClO_11_	440.1085	[M + Na]^+^	463.1416	0.93
29	17.117	8-*O*-Acetylshanzhiside methyl ester ^b^	C_19_H_28_O_12_	448.1581	[M + Na]^+^	471.1484	1.01
30	18.231	7-*epi*-Loganin ^a^	C_17_H_26_O_10_	390.1526	[M + Na]^+^	412.9987	−3.48
31	18.763	Phlorigidoside A/Phlorigidoside B ^a,c^	C_19_H_28_O_13_	464.153	[M + Na]^+^	487.2436	2.07
32	19.071	7,8-Dehydropenstemonoside ^a^	C_17_H_20_O_10_	388.1369	[M + Na]^+^	411.2286	2.47
33	19.229	Zaluzioside ^a^	C_17_H_24_O_11_	404.1319	[M + Na]^+^	427.1821	1.41
34	19.270	8-epideoxyloganic acid ^a^	C_17_H_24_O_11_	404.1319	[M + Na]^+^	427.1221	−0.04
35	19.520	3,4-dihydroxyphenylethanol-8-*O*-[4-*O*-transcaffeoyl-*β*-d-apiofuranosyl(1→3)-β-d-glucopyranosyl-(1→6)]-*β*-d-glucopyranoside ^a^	C_34_H_44_O_20_	772.2426	[M − H]^−^	771.3347	1.72
36	21.024	Loganin ^a^	C_17_H_26_O_10_	390.1526	[M + Na]^+^	413.1342	−0.20
37	22.292	Forsythoside C/Campneoside II ^a,c^	C_29_H_36_O_16_	640.2003	[M − H]^−^	639.2024	0.16
38	20.699	Echinacoside ^a^	C_35_H_46_O_20_	786.2583	[M − H]^−^	785.3854	1.72
39	22.732	Forsythoside C/Campneoside II ^a,c^	C_29_H_36_O_16_	640.2003	[M − H]^−^	639.0087	−2.87
40	24.043	Forsythoside B ^a^	C_34_H_44_O_19_	756.2477	[M − H]^−^	755.1098	−1.72
41	24.651	Luteolin-7-*O*-*β*-d-glucopyranside ^b^	C_21_H_20_O_11_	448.1006	[M − H]^−^	447.1927	2.24
42	24.906	Isoverbascoside ^a^	C_29_H_36_O_15_	624.2054	[M − H]^−^	623.3075	1.77
43	25.434	Leuteolin-7-*O*-[*β*-d-apiose(6→1)]-β-glucoside ^a^	C_26_H_28_O_15_	580.1428	[M − H]^−^	579.3349	3.45
44	27.441	Verbascoside ^a^	C_29_H_36_O_15_	624.2054	[M − H]^−^	623.0175	−2.89
45	28.743	Apigenin-7-*O*-*β*-d-glucopyranoside ^a^	C_21_H_20_O_10_	432.1056	[M − H]^−^	431.1277	0.70
46	29.711	Crenatoside/Orobanchoside ^a^	C_29_H_34_O_15_	622.1898	[M − H]^−^	621.0619	−1.93
47	31.419	Leucosceptoside A ^a,c^	C_30_H_38_O_15_	638.2211	[M − H]^−^	637.1132	−1.57
48	31.577	Lamiophlomioside A ^a^	C_36_H_48_O_19_	784.279	[M − H]^−^	783.3216	0.65
49	31.419	Tenuifoliside C ^a^	C_35_H_44_O_19_	768.2476	[M − H]^−^	767.1397	−1.30
50	31.577	Luteolin-7-*O*-*β*-d-(6-O-acetate)-glucopyranoside ^a^	C_21_H_30_O_13_	490.1686	[M − H]^−^	489.2207	1.23
51	35.924	Apigenin7-*O*-(6”-(E)-p-coumaroyl-β-d-galactopyran-osid ^a^	C_30_H_26_O_12_	578.1424	[M − H]^−^	577.0445	−1.56

^a^ Identified by the reference compounds reported previously in *L. rotata* and *Lamium* species. ^b^ Identified these compounds with standards. ^c^ Compounds were found in this plant for the first time.

**Table 3:** The origins of the materials and GenBank accession numbers of ITS2 sequences.

No.	Species	Sources	GPS Coordinates		GenBank Accession Number
L1-7	*L. rotata*	MaQu and LuQu county in Gansu	E:101°	W:33°	KP699732-34, 36, 44, 38-39
L8-14	*L. rotata*	BianBa, LeiWuQI, NaQu, BiRu BaSu county in Tibet	E:93°	W:31°	KP699743/45-4750-51/54
L15-21	*L. rotata*	DeGe, ShiQu and SeDa, county in Sichuan	E:102°	W:32°	KP699753, 56-61
L22-25	*L. rotata*	ZhiDuo, JiuZhi and HeNan county in Qinghai	E:99°	W:34°	KP699735, 37, 42, 55
A1-7	*A. ovalifolia*	Hongyuan, SeDa and DeGe county in Sichuan	E:102°	W:32°	KP699723-29
O1	*O. wartii*	GenBank	-	-	KY771016-23

## Supplementary Materials

Supplementary File 1
