# Supplementary figures and images for: Lamiophlomis rotata Identification via ITS2 Barcode and Quality Evaluation by UPLC-QTOF-MS Couple with Multivariate Analyses
Source: Molecules. 2018 Dec 11;23(12):3289. doi: 10.3390/molecules23123289 (PMC6320854; doi:10.3390/molecules23123289)

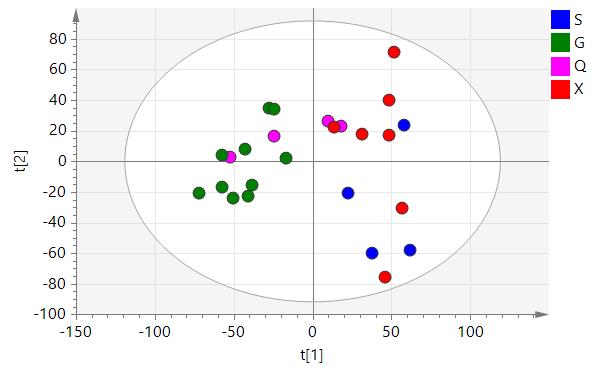

Supplement: Supplementary File 1 [file molecules-23-03289-s001.jpg]
